# Sanitary Features of the Present Campaign

**Published:** 1915-03-20

**Authors:** 


### THE WAR AND THE WOUNDED.

### Sanitary Features of the Present Campaign.

A large gathering assembled in the hall of the Royal Society of Arts on Friday, March 12, to hear a lecture on "The War and the Wounded" by Mr. Adolphe Smith, sanitary commissioner of the *Lancet*, who served in the French ambulances during the siege of Paris.

The chair was occupied by Sir William J. Collins, K.C.V.O., M.D., M.S., who stated that the lecture was being delivered under the joint auspices of the Chadwick Trust and the League of Mercy, a perfectly natural association, seeing that the first of these sought to inculcate right-thinking in regard to matters of health and disease, while the League of Mercy, by its beneficent services to voluntary hospitals, translated the teaching of sanitary science into the kindly ministrations to the sick in those hospitals.

The lecturer said it was in the sequence of events that history taught the most useful lessons, and reminded his hearers of the events which led up to the siege of Paris, the main factor in which was the fact that, for political reasons, the French Army dare not retire on Paris, where it would have joined another army. Consequently it was surrounded at Sedan. He spoke of the need in war-time of an absolute truce of parties. He was in Paris during the whole of the siege, and spoke of the inhabitants eating first decrepit cab-horses, then more succulent thoroughbreds. But later there was no bread or fuel nor any horses, dogs, cats, nor animals at the Zoo; all had been eaten. He had seen a single carrot sold for 3s. and a slice of beef for 10s., while a rabbit realised £2. Rats were plentiful for a time, and the street arab gave to them the name "rabbits of the siege." The winter of 1870-1 was very severe; there was bad food, absence of sanitation and of fever hospitals. During one week of the siege there were 800 deaths from small-pox alone, while in a single week there were, among the civilian population, 6,000 deaths. Dysentery was one of the most common diseases, due largely to the improper and insufficient food. The lecturer proceeded to give some humorous manifestations of "spy mania" in Paris at that time. He thought the old system of organisation of the ambulance work in the French Army, under business men, was preferable to the present scheme of control by doctors, whose education had not embraced training in the requirements of the carrying services. In the present war defects in this branch of the French Army had been very marked. During the Franco-German War the German troops were considered to be more healthy than in any previous war, and yet there were 400,000 deaths from sickness, compared with 100,000 from wounds. Our troops in the present campaign had, so far, been comparatively free from sickness. In Paris a great lesson was taught by the American ambulance. The Americans in Paris could not obtain a building to house the wounded in their charge; they therefore built a huge canvas tent with a wooden floor, and stoves burning in the centre. More lives were saved in that tent, in proportion, than in the other hospitals, and it was found to be due to the excellent ventilation without draught afforded by the canvas walls.

In the present war the great distinguishing feature was the absence of any sort of truce or armistice, so that the recovering of the wounded had to be carried out at night. Another evil feature of the present war was the increased danger of septic poisoning, due to remaining for long periods in the trenches, especially in such thickly manured localities. And in the heavy rains which had been experienced there of late it had been impossible to prevent an occasional overflow of the latrines and the consequent contamination of the trenches. Typhoid fever had been very rife among the Belgian troops, and the Red Cross Society had voted £10,000 to meet the emergency. At the beginning of the war there was an insufficient supply of drugs, especially disinfectants, and a great proportion of the rubber goods had been supplied by Germany. Even the French surgical instruments were sent in the rough from Germany, and were only finished in Paris. He extolled the French system of baths and spraying machines, which could be taken from house to house. The great need at the present time was prompt aid in removing the wounded from the battlefield. There were inventions by means of which men could be operated upon almost at the firing line; and barges had been converted into hospital wards, so that travelling did not involve the jolting which was inseparable from road conveyance. There had also been provided motor vehicles with very good springs. There was a great need for more ambulance trains. He spoke with much pride of the help which so many English people had rendered to the Belgians at their time of supreme need. Within two or three days 12,000 Belgian wounded were landed in England.

He concluded by calling attention to the great activities of the British hospitals and the need for everyone's help, not only in caring for those stricken in body, but also for those who were afflicted in mind and required all the care and sympathy which could be extended to them.

The lecture was illustrated by an instructive series of slides.

